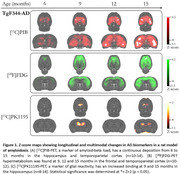# Longitudinal Changes in Alzheimer's Disease Imaging Biomarkers in a Genetically Modified Rat Model of Amyloidosis

**DOI:** 10.1002/alz70855_105561

**Published:** 2025-12-24

**Authors:** Gabriela Lazzarotto, Lidia Emmanuela Wiazowski Spelta, Cleinando Clemente da Silva Vera, Andreia Rocha, Débora Guerini de Souza, Daniele de Paula Faria, Eduardo R. Zimmer

**Affiliations:** ^1^ UFRGS, Porto Alegre, Brazil; ^2^ USP, São Paulo, Brazil; ^3^ University of Pittsburgh, Pittsburgh, PA, USA

## Abstract

**Background:**

Positron emission tomography (PET) radiotracers targeting amyloid‐beta (Aβ) plaques, glucose metabolism, and glial reactivity can be used to diagnose and monitor Alzheimer's disease (AD) progression over time. However, whether animal models recapitulate the temporal progression of AD biomarker abnormalities is still unclear. This study aimed to longitudinally evaluate brain Aβ accumulation, glucose metabolism, and glial responses in a rat model of amyloidosis using PET‐imaging.

**Method:**

Longitudinal PET imaging with [^18^F]FDG, [^11^C]PK11195, and [^11^C]PIB was performed at 6, 9, 12, and 15 months of age in wild‐type (WT) and TgF344‐AD (Tg) rats. Images were manually co‐registered to a rat magnetic resonance imaging template. Standardized uptake values (SUV) were calculated for [^18^F]FDG and [^11^C]PK11195, while SUV ratios (SUVR) for [^11^C]PIB were calculated using the pons as the reference region. Results were normalized using Z‐scores, with statistical significance defined as Z > 2 (*p* < 0.05).

**Result:**

No significant differences were observed between wild‐type (WT) and transgenic (Tg) animals at 6 months. However, by 9 months, Tg animals exhibited increased amyloid deposition (max Z‐value=3.9, entorhinal cortex), enhanced glucose metabolism (max Z‐value=5,1, frontal cortex), and heightened glial reactivity (max Z‐value=2,7, hippocampus). At 12 months, the levels of Aβ load (max Z‐value=5.1, entorhinal cortex) and glucose hypermetabolism (max Z‐value= 3.7, frontal cortex) continued to rise, while glial reactivity remained unchanged. By 15 months, Aβ load increased even further (max‐Z value=5.9, entorhinal cortex), glucose metabolism stay elevated (max Z‐value= 4.9, frontal cortex), and glial reactivity showed another increase (max Z‐value=2.7, hippocampus).

**Conclusion:**

These findings suggest that during the early stages of Aβ deposition, there is a simultaneous increase in glucose metabolism and glial reactivity. This is followed by a transient decrease in glial reactivity, with a resurgence of glial activation in later stages, while glucose metabolism remains elevated. This supports the hypothesis that amyloid deposition triggers both early and late neuroinflammatory responses. The persistent hypermetabolism observed reinforces that murine models are more resilient to amyloid pathology. These results underscore the value of an imaging platform to study the temporal progression of amyloid pathology in genetically modified rodent models with strong translational relevance.